# Supplementary figures and images for: A proximity-based in silico approach to identify redox-labile disulfide bonds: The example of FVIII
Source: PLoS One. 2022 Feb 7;17(2):e0262409. doi: 10.1371/journal.pone.0262409 (PMC8820644; doi:10.1371/journal.pone.0262409)

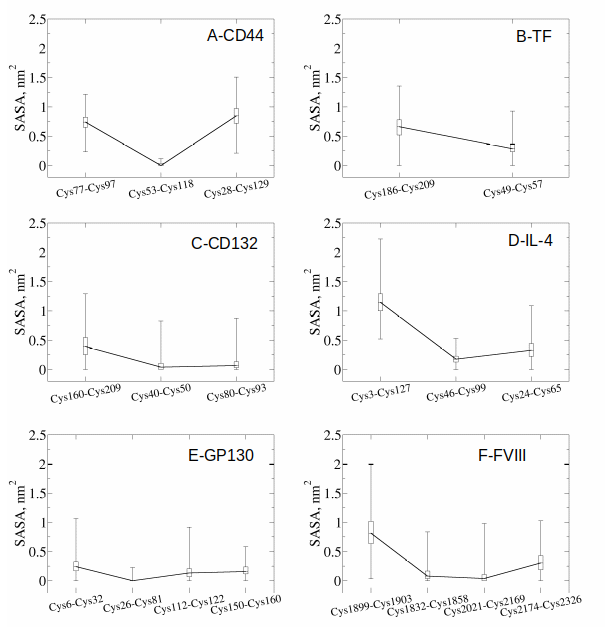

Supplement: S1 Fig — Boxplots showing the solvent accessible surface area (SASA) of the disulfide-forming cysteines during the MD simulations. The different panels refer to CD44 (A), tissue factor (B), CD132 (C), IL-4 (D), the D1 and D2 domains of GP130 (E), and FVIII light chains (F). In the boxplots, the top bar is the maximum observation, the lower bar is the minimum observation, the top of the box is the third quartile, the bottom of the box is the first quartile, while the middle bar represents the median. The medians are connected by a straight line to guide the eye. (TIF) [file pone.0262409.s001.tif]

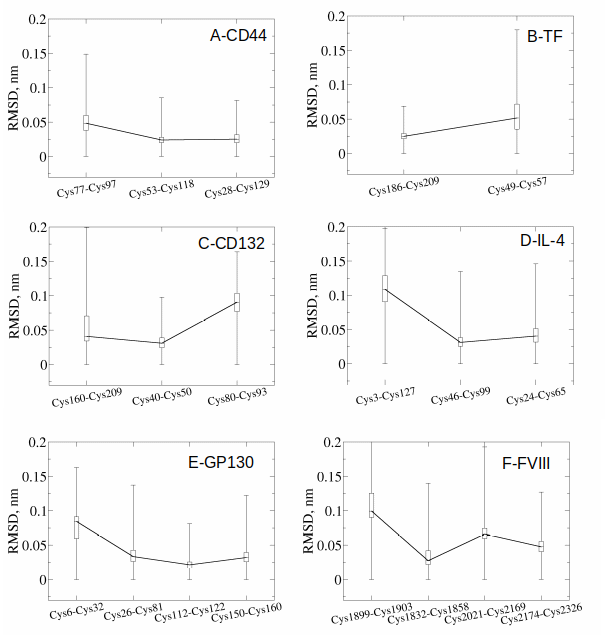

Supplement: S2 Fig — Boxplots showing the root mean square deviation (RMSD) of the disulfide bonds during the MD simulations. The different panels refer to CD44 (A), tissue factor (B), CD132 (C), IL-4 (D), the D1 and D2 domains of GP130 (E), and FVIII light chains (F). In the boxplots, the top bar is the maximum observation, the lower bar is the minimum observation, the top of the box is the third quartile, the bottom of the box is the first quartile, while the middle bar represents the median. The medians are connected by a straight line to guide the eye. (TIF) [file pone.0262409.s002.tif]

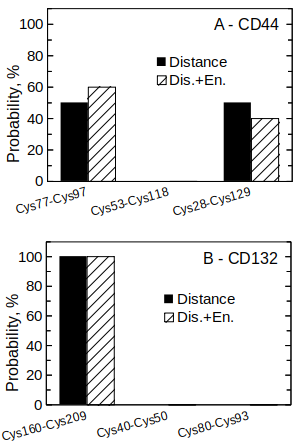

Supplement: S3 Fig — Histograms showing the probability that each of the (A) CD44 or (B) CD132 disulfide bonds is reduced by DTT according to the distance- (plain black bars) or distance+energy-based (dashed bars) criteria. (TIF) [file pone.0262409.s003.tif]

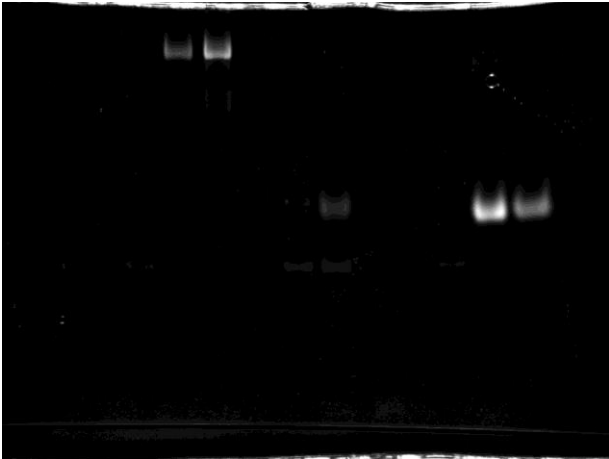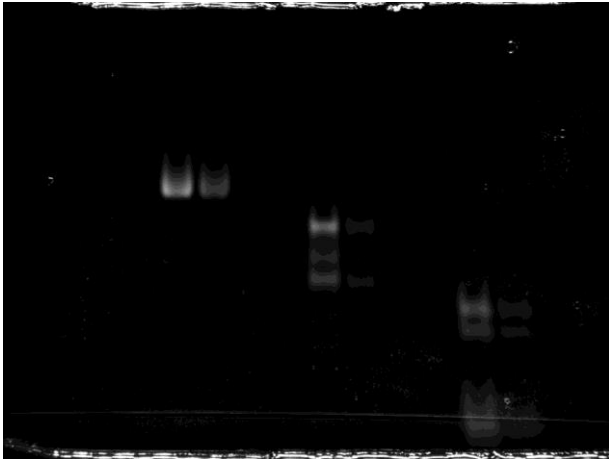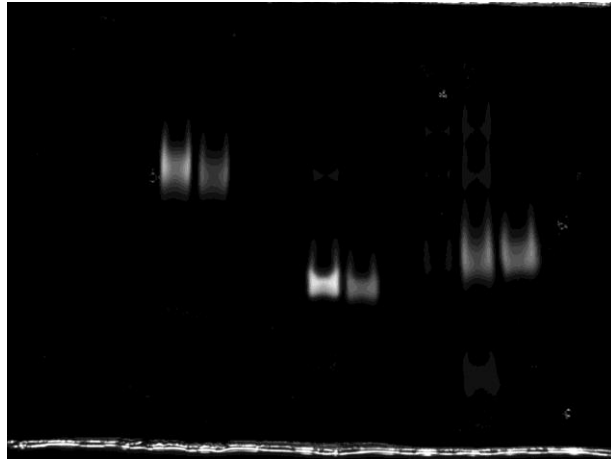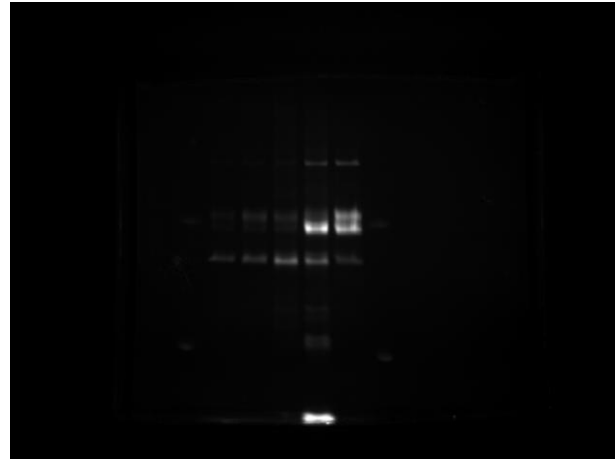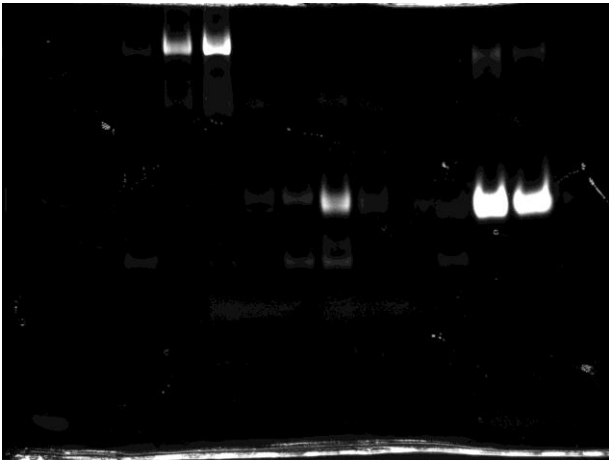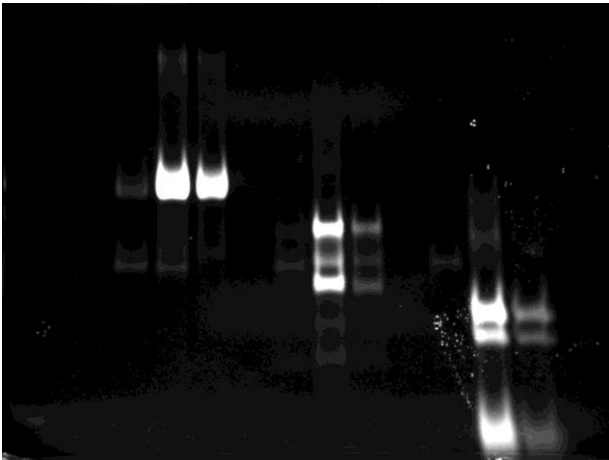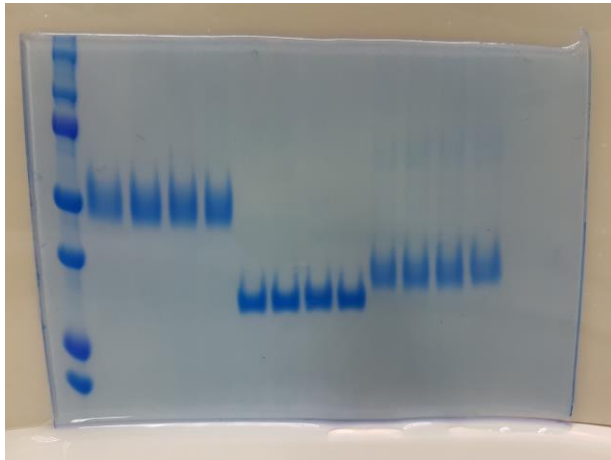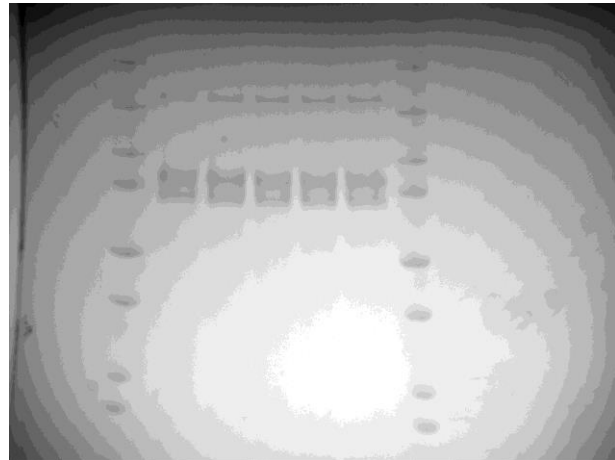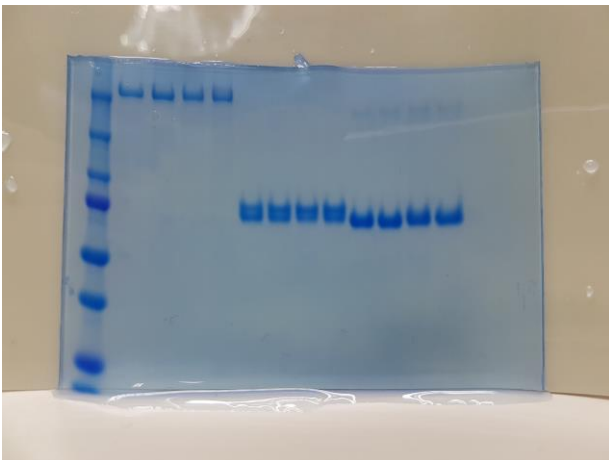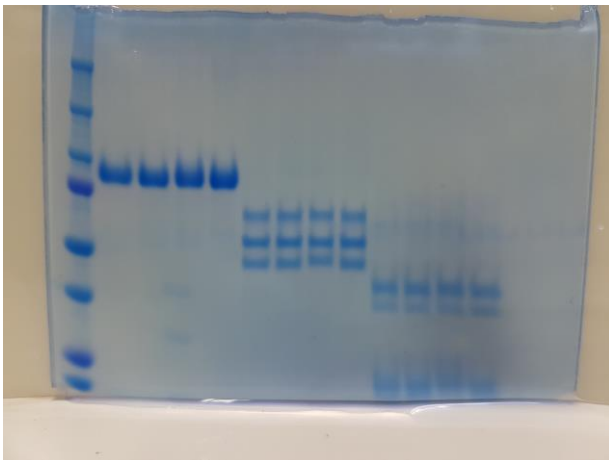

Gels for Figure 2

## Gels for Figure 8

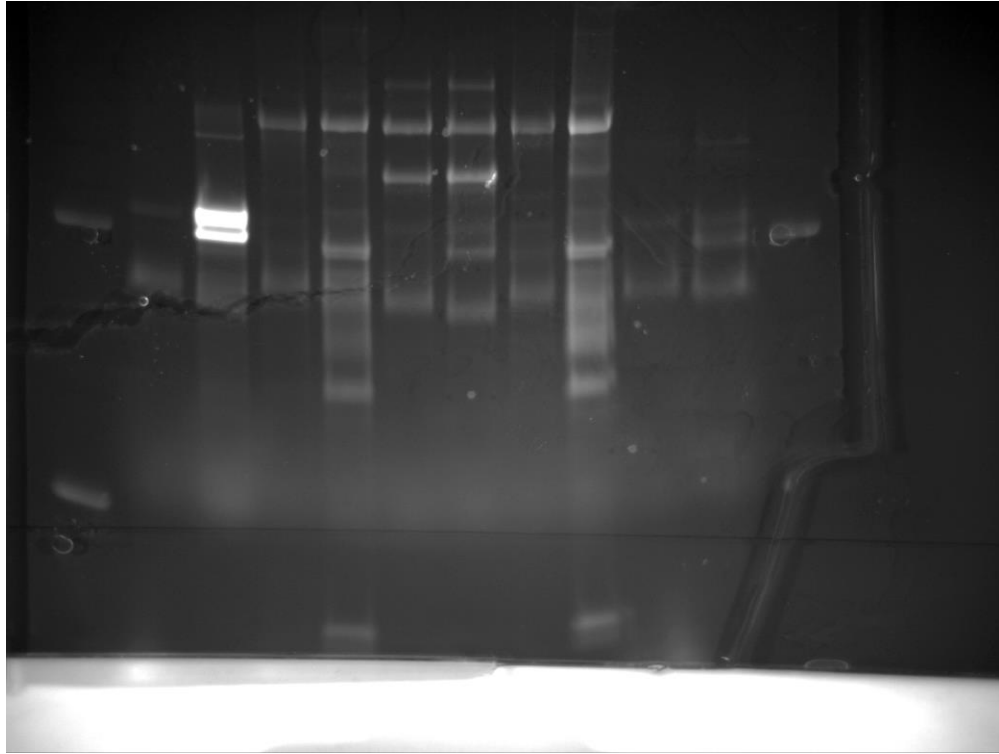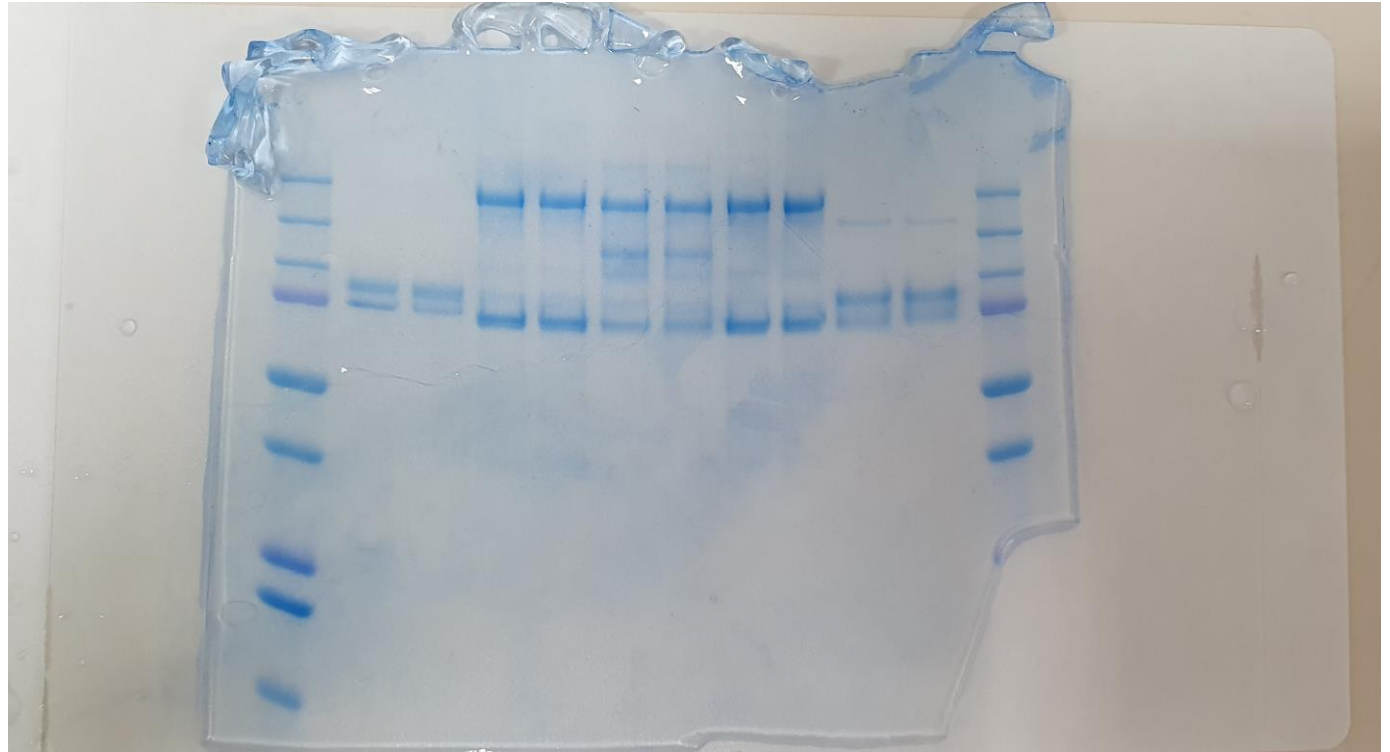

Supplement: S1 Raw images — (PDF) [file pone.0262409.s005.pdf]
