# Supplementary material for: A proximity-based in silico approach to identify redox-labile disulfide bonds: The example of FVIII
Source: PLoS One. 2022 Feb 7;17(2):e0262409. doi: 10.1371/journal.pone.0262409 (PMC8820644; doi:10.1371/journal.pone.0262409)
Supplement: Attachment — Submitted filename: review 2 comments.pdf [file pone.0262409.s006.pdf]

In "A proximity-based in silico approach to identify redox-labile disulfide bonds: the example of FVIII", Arsiccio and coworkers developed a MD protocol to identify labile disulfide bonds (DBs) in a small set of proteins with known structure, and then extrapolate the method and experimentally validated it by studying the redox-mediated effects of the coagulation factor VIII (FVIII). The agreement observed between the simulations and experimental evidence for the FVIII particular case is remarkable and well supported. Although Im not an expert, the significance of the biological problem regarding the role of labile disulfide bonds in the coagulation process is indubitable. However, the work is centered on the development of the MD protocol, and the score used by the authors is not validated and/or described-discussed enough to justify its future use in a broader sense. So, I do not recommend the publication of this work in its actual state. General and specific points to asses for future versions are described below.

General points:

1)As stated before, my main concern regarding this research, is the validity and convenience of the developed protocol to answer the DBs lability question. The authors have picked a proximity-based criterium using a distribution related parameter somehow indicating the local concentration of a probe (TCEP) near the DBs, that allow them to order (not identify unambiguosly) the lability of different DBs within a protein, in a small set of selected cases. In this sense, different topics should be addresed:

- A simple visual inspection of the DBs selected to study (Fig 2), led to the impression that DBs accesibility to any molecule (including solvent molecules) is a very important property to determine a particular DB lability (in terms of chemical tendency to get reduced). This idea is not new at all, the authors even discussed it when comparing the SASAs of the FVIII DBs (Table 2), please see these references in depth: https://doi.org/10.1016/j.bpj.2014.06.025; https://doi.org/10.1098/rsos.171058. Furthermore, a simple calculation of the DBs SASA's from the X-ray structures used by the authors suggest the same trend presented by the authors using the MD protocol:

protein	pdb	disulfide	SASA (A2)
CD44	1uuh (chain A)	77-197	53,4
		53-118	1
		28-119	40,2
TF	1boy	186-209	36,1
		49-57	33
CD132	2erj (chain C)	160-209	18,1
		40-50	10,9
		80-93	0,7
IL-4	3bpl (chain A)	3-127	101,7
		46-99	12,6
		24-65	8,4
GP130	1p9m (chain A)	6-32	46,2
		26-81	2,7
		112-122	8
		150-160	13,4

So, at this point I do not see the need of such complex metadynamics MD simulations to get an indicator of DBs lability. The authors need to justify much more deeply the use of the simulation protocol, identifying the advantages of such a use.

- It is my opinion that the validation of the MD protocol for it use in any DBs containing protein should include more DBs/protein examples, more protein folding types and DBs environments.

- The use of TCEP as the probe for the proximity criterium is based on its well known use as reductant. However, proximity probabilities arising from the MD simulations does not necesarly be related with reactivity. Added to the fact that the protocol is highlighting most solvent accesible DBs, the question that arises is: would any probe serve for the proximity criterium? In other terms, what are TCEP properties that justify its use? This aspect should be better discussed.

- Related with the previous point, the authors made use only of TCEP not only as the probe for the MD protocol, but also as non-physiological reducing agent. As authors may know, the characteristics of different reductants result in different reduction yields and specificities. For example, this fact is very well discussed in 10.1006/abio.1999.4203 for a DTT/TCEP comparison. So, I suggest to include at least DTT in the mass spectrometry and activity assays, in order to avoid possible biases.

- If maintening TCEP proximity criterium, I would advice to show the distributions in other fashion, boxplots maybe, as histograms as showed in this version does not seem to be the better/simpler way to compare the results between different DBs. In fact, they are not shown with the same bin width criterium for every system

2) Regarding the compartive study of FVIII dynamics and conformational changes upon C1899-1903 DB reduction: it seems to me that 100 ns of conventional MD is not enough sampling to characterize this phenomena, mainly because of the initial structure bias present for the reduced system. Although I acknowledge that the size of the system presents a limitation to achieve more exhaustive sampling, there are some strategies that can overcome this issue at least partially. I would advise to characterize much better the dynamic properties of the two systems, maybe by the use of some replica of accelerated MDs followed by conventional MDs or any other enhanced sampling method that the authors are familiar with. The global properties presented does not seem to be statistically well sampled, and so local properties such as HBs in particular protein regions would also present a big sampling bias.

Specific points:

- Through the document, there are several issues regarding references and the use of some reference manager software, please take care of it.

- There are also abbreviations and acronysms not defined previously, such as FVIII in the Abstract, DSB in the text…

- In the Introduction, there are some issues with capital letter use

- The format in which DBs are referred through out the text and figures is ambigous (Cys#-Cys#, Cys # - Cys #, Cys #-#, #-# disulfide bond...), I would advise to define and use one formal only

- Figure 5B is very low quality. Also, it is not easy to understand the reason why Figures 5B and 5C are not highlighting exactly the same regions of the protein, this question can be extended to Figure

6A. It seems that all 3 figures (5B, 5C and 6A) are meant to show basically the same observed changes, so I would only use Figure 5C with a clear representation of the scale that is being used.

- The definition of the different FVIII products in Figure 8 is not easy to understand and also is not clarified in the Figure caption.
