# Supplementary material for: A proximity-based in silico approach to identify redox-labile disulfide bonds: The example of FVIII
Source: PLoS One. 2022 Feb 7;17(2):e0262409. doi: 10.1371/journal.pone.0262409 (PMC8820644; doi:10.1371/journal.pone.0262409)
Supplement: Attachment — Submitted filename: reply-letter.docx [file pone.0262409.s008.docx]

**A proximity-based *in silico* approach to identify redox-labile disulfide bonds: the example of FVIII**

*Andrea Arsiccio, Clive Metcalfe, Roberto Pisano, Sanj Raut, and Carmen Coxon*

The authors would like to thank the reviewers for their accurate and fruitful revision of the present manuscript. Here, the point-by-point reply to reviewers’ comments is given, while changes made into the manuscript have been highlighted as colored text.

**REVIEWER #1**

GENERAL COMMENTS: The new version of "A proximity-based in silico approach to identify redox-labile disulfide bonds: the example of FVIII" has been significantly improved compared to the previous one. The authors have included new simulations and analyses. From my viewpoint, the paper is a good contribution to study proteins and disulfide bond lability.

ANSWER TO GENERAL COMMENTS: We thank the reviewer for the revision of the present manuscript, which allowed us to improve our work.

**REVIEWER #2**

GENERAL COMMENTS: The revised version of the work by Arsiccio and coworkers is very much improved and several issues previously highlighted were corrected and/or clarified. Importantly, the computational methodology is now much more clear together with the relevance of the use of such a "proximity based" approach using TCEP as the probe. Nevertheless, some points are still not correctly addressed, so I would suggest to discuss and/or correct them in future versions.

ANSWER TO GENERAL COMMENTS: We very much appreciate the constructive comments of the reviewer. We have tried to address the comments raised by the referee, and hope the new version meets criteria for publication.

SPECIFIC COMMENTS

COMMENT #1: Although I think it is an editorial responsibility to evaluate the implicances regarding restrictions to perform new experiments in these COVID times, I do think that experimental controls such CD (as suggested by reviewer 1) and/or MS and activity assays regarding the use of DTT are important for the present work

Author reply #1:

COMMENT #2: I agree the SASA measurements of static structures is "not always completely representative of the protein dynamics", however, is still a very good initial approximation of the property that is been measured, as highlighted in the previous review. That's the reason behind the suggestion of measuring SASA from the MDs, which as shown now in Fig S1, correlates well (obviously not perfectly) with DBs lability.

Author reply #2: The reviewer is right, and we appreciated the suggestion of measuring SASA from the MD simulations. A comment on this has been added to the revised manuscript,

“The average SASA of the different disulfide bonds correlates fairly well with their lability (see Figure S1), and is therefore a good initial approximation.”

COMMENT #3: Although the authors made an effort to enlarge the statistical significance of the changes observed after reducing Cys1899-Cys1903 in FVIII, I still found the conclusions a little far-stretched, and much better sampling of the systems is needed. The author's arguments against the use of REMD may be reasonable and depends on the available computational capabilities, but how about accelerated MD? 200 ns long MDs does seem to be enough to explain allosteric (long distance) conformational changes of DB reduction in such more than a thousand amino acids protein. I am not saying what is being observed is way wrong, my reasoning is that the dynamical behaviour is extremely biased by the initial structure, and the same is valid for any arising property. Thus, I sincerely think that if this issue is important for this particular research piece, much more sampling effort needs to be performed.

Regarding this point, Figure 6 needs some work:

i. RMSF is not informative at all

ii. It is quite difficult to extract information from FIg 6C. Taking aside the fact that the dynamical properties are not well sampled, Q parameter is an statical descriptor, which is also very dependent of protein alignment. Maybe this ref would be useful (10.1371/journal.pone.0119264). Also, I would suggest to align domains separately for these kinds of analysis, as the large size of the system sometimes difficult adequate alignment.

iii. Through out the text and this figure, the terms "disulfide present" and "disulfide broken" does not seem to be chemically correct, please consider to use oxidized and reduced.

Author reply #3: We understand the concerns of the reviewer. To give an idea of the computational cost required for REMD simulations of FVIII, if we aimed at using temperatures in the range 300-500 K, with an exchange probability between 0.2 and 0.3 (which is generally desired), according to the protocol described in (Alexandra Patriksson and David van der Spoel, A temperature predictor for parallel tempering simulations Phys. Chem. Chem. Phys., 10 pp. 2073-2077 (2008) <http://dx.doi.org/10.1039/b716554d>) we would need approximately 350 replicas. For each replica, we would need at least 31341 core hours to run 100 ns, which means that more than 10 million core hours would be required for the whole REMD simulation. This is only a rough estimate, and the computational cost may actually increase, because FVIII would denature at high temperatures, meaning that we would need to increase our box size and, consequently, the number of replicas. Unfortunately, such a high computational cost makes REMD simulations impracticable. For what concerns other accelerated MD simulations, such as umbrella sampling or metadynamics, they would require the choice of one (or more) collective variables to enhance the transition. Unfortunately, the choice of such collective variables is not easy when structure equilibration is desired. Generally, collective variables are used with the objective to push the system away from minima (for instance forcing the folding/unfolding transition) while we here aim at sampling as much as possible the energy minimum. For this reason, we would prefer not to try with enhanced sampling methods based on the biasing of collective variables, as we may risk pushing the system even further from the desired minimum. However, the reviewer is right about a possible bias in the results due to the initial structure, and we added a comment to the manuscript to warn the readers that, due to the very large size of FVIII, enhanced sampling methods were impracticable and the results of the conventional MD simulations presented may suffer from imperfect sampling.

“It is important to note that enhanced sampling methods were impracticable for these simulations due to the very large size of FVIII, and we therefore conducted conventional MD runs. We extended the simulation time until the computed properties (radius of gyration, internal hydrogen bonding network, solvent accessible surface area and backbone RMSD compared to the crystal structure, as shown in Figure 6) converged to stable values. The results of the conventional MD simulations presented are therefore a good indication of what may happen immediately after reduction of the labile disulfide bond; however, because of the impossibility to use enhanced sampling techniques, the reader should be aware that they may be not completely representative of long-time shifts in the protein conformation.”

As for the other comments,

i. we have removed the RMSF from Figure 6

ii. we agree with the reviewer that the Q parameter is a statical descriptor, but we applied it to the most sampled conformations in the equilibrated trajectories, as extracted by means of the Daura algorithm (Ref. 51 in the text). Such conformations are representative of more than 80% of the equilibrated trajectories, making the considerations drawn from the alignment more informative. However, as suggested by the reviewer, we also separately aligned the different domains of FVIII (new Figure 6C in the revised manuscript).

iii. We thank the reviewer for this comment, we modified the notation throughout the manuscript.

MINOR POINTS:

1. Colouring of FVIII protein in Figure 1 is different from the other systems

2. Please consider to change the term "normal MD" to "conventional MD"

3. Separating ticks in x-axis of Fig 4 and Fig S3 are somehow misleading...also 0 and 100 values are difficult to observe in those graphs, please consider reviewing them, maybe changing scales

REPLY TO MINOR POINTS:

1. We thank the reviewer for pointing this out. The colouring of FVIII has been changed.

2. The term ‘normal MD’ has been changed to ‘conventional MD’ as suggested.

3. Figs. 4 and S3 have been modified as recommended.
